# Comparison of open and closed reduction and percutaneous pinning for pediatric lateral humeral condyle fractures: A systematic review and meta-analysis

**DOI:** 10.1097/MD.0000000000042060

**Published:** 2025-04-11

**Authors:** Xinxin Xu, Jicheng Zeng, Kai Wang, Qiyu Meng, Si Yuan, Jiangtao Shen, Zhanchun Li, Xitao Sun

**Affiliations:** a Department of Orthopedics, Zhejiang Cancer Hospital, Hangzhou, China; b Shenzhen Baoan Air Sea Hospital, Shenzhen, China; c The First Clinical Medical College of Zhejiang Chinese Medical University, Hangzhou, China; d Department of Orthopedics, The First Affiliated Hospital of Zhejiang Chinese Medical University (Zhejiang Provincial Hospital of Traditional Chinese Medicine), Hangzhou, China.

**Keywords:** closed reduction and percutaneous pinning, lateral condyle fractures of humerus, meta-analysis, open reduction and percutaneous pinning

## Abstract

**Background:**

Open reduction and percutaneous pinning (ORPP) is commonly regarded as the primary treatment option for serious displaced lateral condyle fractures of the humerus (LCFs) in children. However, some authors have suggested that closed reduction and percutaneous pinning (CRPP) may be an appropriate method for treating LCFs. This meta-analysis aims to compare the outcomes of these 2 fixation techniques.

**Methods:**

Our study conducted a search of the Pubmed, Embase, and Cochrane Library databases for published research up to October 1, 2022. Our analysis comprehensively compared the operation failure rate, elbow function, and complication rate between CRPP and ORPP. This study was registered with PROSPERO (CRD42022379655).

**Results:**

Our analysis included 6 non-randomized controlled trials and 532 patients. We used the Newcastle Ottawa Scale to assess the bias risk of these studies, with scores ranging from 6 to 9. The results indicate that both CRPP and ORPP yielded satisfactory elbow function outcomes (OR = 0.35, 95% CI = 0.07–1.88, *P* = .22). However, CRPP had a significant rate of operative failure (17.65%, OR = 21.77, 95% CI = 3.98–119.08, *P* = .0004) but a lower likelihood of unsightly scars (OR = 0.06, 95% CI = 0.01–0.31, *P* = .008). The failure rate of surgery is 0% in ORPP. There were no significant differences found in total infection (OR = 0.46, 95% CI = 0.21–1.01, *P* = .05), avascular necrosis (OR = 0.84, 95% CI = 0.09–7.79, *P* = .88), delayed union (OR = 1.49, 95% CI = 0.06–37.35, *P* = .81), or surgical time (MD = 4.46, 95% CI = −25.92 to 34.84, *P* = .77).

**Conclusions:**

In comparison to ORPP, CRPP may result in a higher rate of operative failure but has been found to significantly reduce the occurrence of unsightly scars. Both CRPP and ORPP showed similar levels of postoperative functional satisfaction, with no statistical difference in other complications. Our research suggests that qualified closed reduction is a viable option for doctors to treat LCF.

**Levels of evidence:**

IV.

## 1. Introduction

Lateral condyle fractures of the humerus (LCFs) are the second most common type of elbow joint fracture in children, accounting for approximately 12% to 20% of all elbow injuries.^[1–4]^ The treatment for LCFs depends on the degree of displacement and stability of the fracture.^[5]^ Non-displaced fractures can be treated conservatively with casting,^[6]^ but surgical intervention is usually recommended for serious displaced fractures with displacement >2 mm, especially those with rotation of fracture fragments.^[7–10]^ LCFs has many complications(such as infections, joint stiffness, nonunion, delayed union, avascular necrosis and so on),^[5–8]^ so it is important for patients to choose the appropriate surgical method. Open reduction and percutaneous pinning (ORPP) has been suggested as the preferred treatment method to achieve consistent reduction of the articular surface and sufficient fracture reduction.^[4–7]^ However, recent studies have reported that closed reduction and percutaneous pinning (CRPP) may be an appropriate treatment option for LCFs.^[11–17]^ Prior studies show that there is no significant difference in complications and prognosis between CRPP and ORPP.^[13–15]^ CRPP offers several advantages over ORPP, including less soft-tissue damage, reduced risk of vascular injury, lower risk of nonunion and avascular necrosis, shorter operation time, and avoidance of unsightly scars.^[18,19]^ The treatment of ORPP can achieve satisfactory anatomical reductionpatients, but it can also develop malunion caused by inadequate reduction or osteonecrosis that is caused by extensive soft-tissue dissection.^[14]^ CRPP treatment enables the attainment of an approximate anatomical reduction and less dissection of soft tissue.^[15]^ There is currently no clear consensus on the use of CRPP for the treatment of LCFs with significant displacement. Therefore, this meta-analysis was conducted to compare ORPP and CRPP for distal humeral lateral condyle fractures in children and provide evidence to guide clinical treatment decisions.

## 2. Materials and methods

### 2.1. Search strategy

Our study conducted a literature search of The Cochrane Library, PubMed, and Embase databases up to October 2022 to compare the effects of ORPP and CRPP on the treatment of LCFs. Our study plan to obtain additional literature from reference lists and exclude non-English languages during the screening process. The detailed and complete search strategy is shown in Table 1. This study was registered with PROSPERO (CRD42022379655).

**Table 1:** The search strategy.

Databases	Search strategy	Results
Pubmed	((((((Humeral Fractures[MeSH Terms]) OR (Fracture, Humeral[Title/Abstract])) OR (Fractures, Humeral[Title/Abstract])) OR (Humeral Fracture[Title/Abstract])) OR (distal humerus fracture[Title/Abstract])) OR (humeral lateral condyle fracture[Title/Abstract])) AND (lateral condyle)	604
Embase	(“distal humeral fracture”/exp OR “distal humerus fracture”:ab,ti OR “humeral condylar fracture”:ab,ti OR “humeral lateral condyle fracture”:ab,ti OR “humeral fractures”:ab,ti OR “fracture, humeral”:ab,ti OR “fractures, humeral”:ab,ti OR “humeral fracture”:ab,ti) AND lateral AND condyle	181
The Cochrane Library	#1 MeSH descriptor: (humeral fractures) explode all trees #2 (fracture, humeral):ti,ab,kw #3 (fractures, humeral):ti,ab,kw #4 (humeral fracture):ti,ab,kw #5 (humeral lateral condyle fracture):ti,ab,kw #6 (lateral condyle) #7 #1 OR #2 OR #3 OR #4 OR #5 #8 #6 AND #7	11

### 2.2. Inclusion and exclusion criteria

Inclusion criteria for this study were: randomized controlled trials (RCTs), prospective or retrospective observational studies (non-RCTs); a comparison of ORPP and CRPP for the treatment of LCFs; the age of the patients in the study is under 14 years old.

Exclusion criteria were: review articles, case reports, commentaries, editorials, letters, animal experiments, cadaveric studies, and technical articles; non-comparative studies; insufficient data such as conference papers and abstract-only publications; studies not published in English; duplicate studies.

### 2.3. Quality assessment

The quality assessment of included RCT studies was carried out by 2 authors in Review Manager 5.4 software, based on the Cochrane Handbook for Systematic Reviews of Interventions. For non-RCT studies, the Newcastle Ottawa Scale (NOS) will be used for assessment.

### 2.4. Data extraction

All selected studies were evaluated by 2 blinded researchers according to inclusion and exclusion criteria. To avoid the risk of bias, 2 independent reviewers reviewed the manuscripts. A third blinded reviewer, belonging to the research group, determined any differences in the selection of the documents. Based on the discussion among the authors, our study designed a table to extract data. Our study extracted data about the first author, publication date, average age, gender distribution, number of patients, type of fracture, surgical time, follow-up period, the failure rate of surgery, elbow function infection (Hardacre criteria), unaesthetic scar, avascular necrosis and delayed union. Any differences were resolved through consultation with a third-party author, if necessary.

### 2.5. Statistical analysis

Statistical analysis was performed using RevMan 5.4 (Cochrane Collaboration, Oxford, UK). For dichotomous outcomes, the results were presented as odds ratios with 95% confidence intervals (CIs). For continuous outcomes, such as operation time, mean differences with 95% CIs were used. Heterogeneity between studies was assessed using the χ^2^ test (with a significance level of *P* < .10) and the *I*^2^ statistic (with a value >50% indicating significant heterogeneity). Pooled results were calculated using a fixed-effects model for values of *P* > .10 and *I*^2^ < 50%, or a random-effects model for values of *P* < .10 and *I*^2^ > 50%.

## 3. Results

### 3.1. Search results

**Figure 1.**
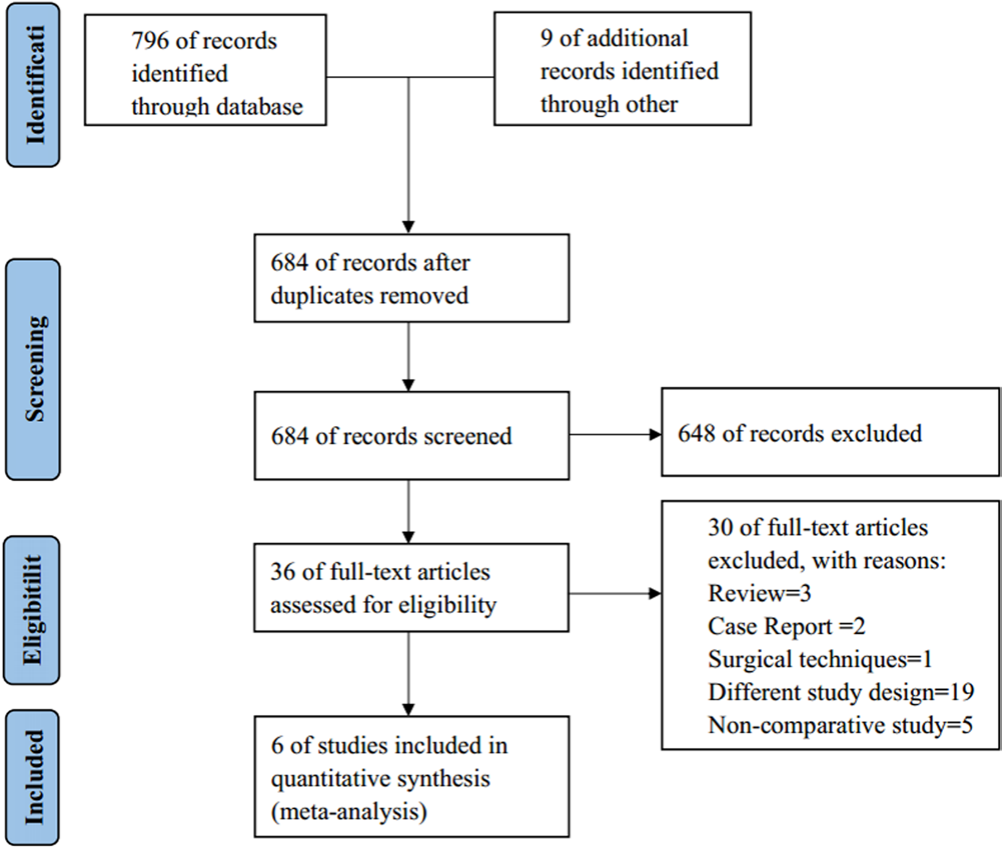
Flow diagram of literature search and selection.

Initially, 796 search results were identified across all 3 databases and 9 of additional literature from reference lists. Our study did not include any gray literature (reports, theses, conference papers, government documents and so on). After removing duplicates, 684 relevant articles remained. The titles and abstracts of these articles were screened, and 36 full-text articles were selected for further review. After applying the inclusion and exclusion criteria, 6 non-RCTs were included in this study.^[12–17]^ Among them, 3 are reviews, 2 are case reports, 1 pertains to surgical techniques, 19 have study designs that do not meet the inclusion criteria, and 5 are non-comparative studies. The research flow chart is presented in Figure 1.

### 3.2. Risk of bias assessment

We conducted a quality assessment of the 6 case-control studies included using the NOS.^[12–17]^ The NOS score range is 0 to 9 points, with 6 to 9 points indicating high research quality. The results showed that 1 study scored 9 points, 3 studies scored 8 points, and 2 studies scored 6 points. 100% of the studies (6/6) scored 6 or above, indicating that the majority of studies performed well in terms of selectivity, comparability, and outcome evaluation. Specifically, all studies scored high in terms of selection, with 66.7% (4/6) of studies scoring 2 out of 2 in comparability, and 16.7% (1/6) of studies scoring 3 out of 3 in outcome evaluation. Due to the high NOS scores in most studies, the results of this study have a high level of credibility. The main issue observed in the included literature was that it primarily consisted of retrospective analyses and did not address the issue of patient follow-up loss. Specific evaluation details are provided in Table 2.

**Table 2:** Assessment of the included cohort studies using the New Castle Ottawa Scale and demographic characteristics.

Author	Selection (4)	Comparability (2)	Exposure (3)	Total score (9)	People (O/C)	Mean age (O/C)	Male patients (O/C)	Fracture style
Justus, 2017	3	1	2	6	141/31	5.29/5.16	64.5%/71.0%	Song (2–5)
Kotb, 2021	4	2	3	9	30/30	6/6.5	83.3%/40.0%	Song (3–5)
Liuqi, 2022	4	2	2	8	31/10	5.39/4.90	54.8%/60%	Song 5
Liwei, 2021	4	2	2	8	62/45	5.1/5.3	66.1%/60%	Song (4–5)
Pennock, 2015	3	1	2	6	51/23	4.5/4.5	64.7%/52.2%	Weiss 2
Yanhan, 2022	4	2	2	8	36/42	5.9/4.4	66.7%/76.2%	JFC3/Song 5

Abbreviations: C = closed group, O = open group.

### 3.3. Study characteristics

A total of 532 individuals from 6 studies were included in this analysis, with 351 in the open group and 181 in the closed group. The sample sizes of all the studies were relatively small, ranging from 41 to 172 patients. Table 2 provides a summary of the demographic characteristics included in the study.

### 3.4. The failure rate of surgery

**Figure 2.**
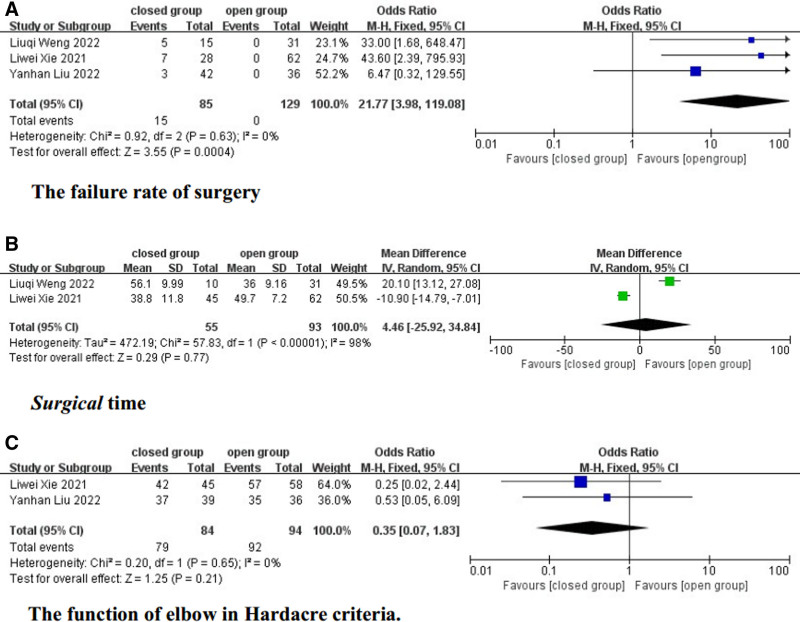
Forest plot of outcome.

Three studies reported failed cases in CRPP.^[15–17]^ After data extraction, our study analyzed the failure rate of CRPP specifically for Song stage 5 LCFs in 3 studies. The results showed that the failure rate of CRPP for Song stage 5 LCFs was approximately 17.65%. In contrast, the failure rate of surgery is 0% in ORPP. At the same time, there was no difference in prognosis among patients who converted from CRPP failure to ORPP in these studies. A fixed-effects model was used, and no significant heterogeneity was observed (*I*^2^ = 0%, *P* = .63). The incidence of failure rate in the closed group was significantly higher than that in the open group (OR = 21.77, 95% CI = 3.98–119.08, *P* = .0004) (Fig. 2a).

### 3.5. Surgical time

Surgical time data were available in 2 articles.^[15,17]^ A random-effects model was applied due to significant heterogeneity (*I*^2^ = 98%, *P* < .00001). The results indicated no significant difference between CRPP and ORPP in terms of surgical time (MD = 4.46, 95% CI = −25.92 to 34.84, *P* = .77) (Fig. 2B).

### 3.6. The function of elbow

Two articles provided evaluation data on elbow function during follow-up.^[15,16]^ The evaluation of elbow joint function yielded an excellent and good rate of 100%. A fixed-effects model was used, and no significant heterogeneity was found (*I*^2^ = 0%, *P* = .65). The rate of excellent elbow function in the open group and closed group was 97.87% and 94.05%, respectively. There was no significant difference in elbow function between CRPP and ORPP (OR = 0.35, 95% CI = 0.07 to 1.83, *P* = .21) (Fig. 2C).

### 3.7. Superficial infection

**Figure 3.**
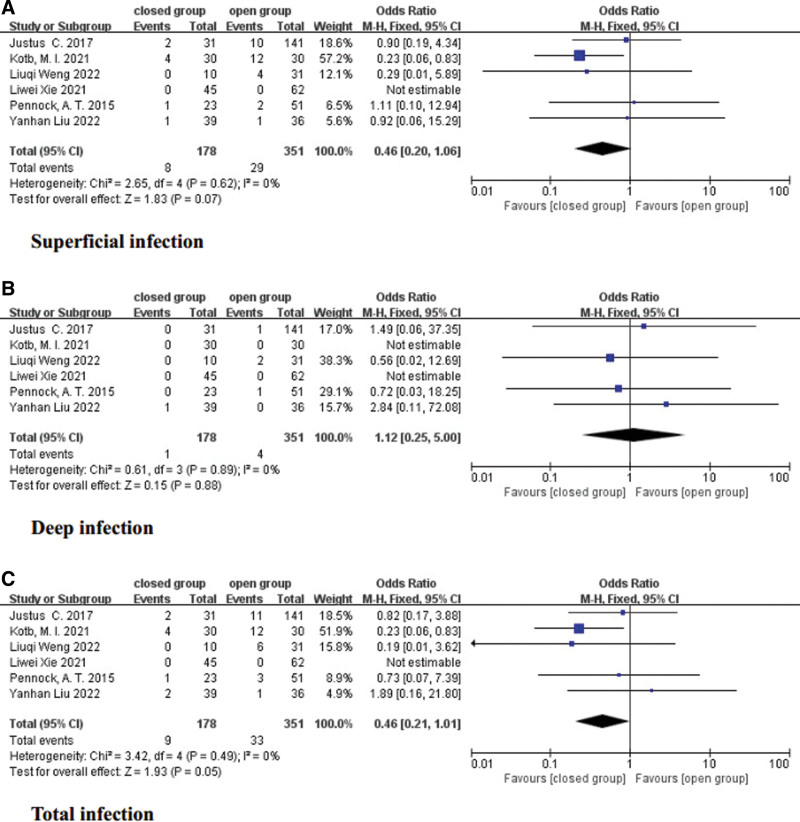
Forest plot of infection.

The incidence of superficial infection was reported in all studies.^[12–17]^ A fixed-effects model was applied, and no significant heterogeneity was observed (*I*^2^ = 0%, *P* = .62). There was no significant difference in the occurrence of superficial infection complications between the open group and the closed group (OR = 0.46, 95% CI = 0.20–1.06, *P* = .07) (Fig. 3A).

### 3.8. Deep infection

The full-text of all studies included information on patients with deep infections.^[12–17]^ A fixed-effects model was utilized, and no significant heterogeneity was found (*I*^2^ = 0%, *P* = .89). The incidence of deep infection complications did not significantly differ between the open group and the closed group (OR = 1.12, 95% CI = 0.25–5.00, *P* = .88) (Fig. 3B).

### 3.9. Total infection

Although the incidence of superficial or deep infection in the closed group was lower than that in the open group, the difference was not statistically significant.^[12–17]^ Therefore, our study conducted an analysis on the total infection rate using a fixed-effects model and found no significant heterogeneity (*I*^2^ = 0%, *P* = .49). The results showed no significant difference in the incidence of total infection complications between the open group and the closed group (OR = 0.46, 95% CI = 0.21–1.01, *P* = .05) (Fig. 3C).

### 3.10. Unaesthetic scar

**Figure 4.**
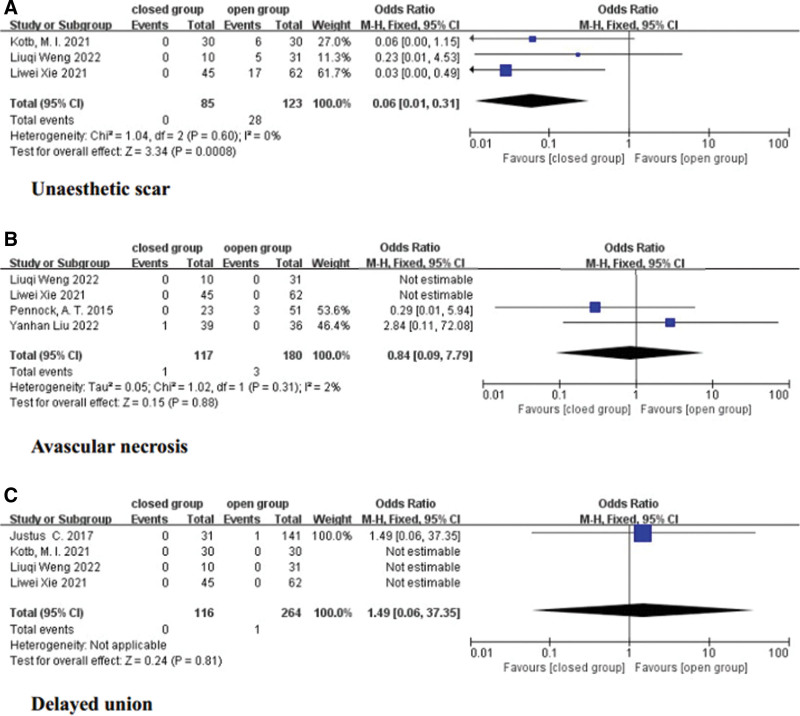
Forest plot of some complications.

Three studies reported the incidence of unaesthetic scars, and a fixed-effects model was applied.^[14,15,17]^ No significant heterogeneity was observed (*I*^2^ = 0%, *P* = .60). The results showed that the incidence of unaesthetic scars was significantly lower in the closed group than in the open group (OR = 0.06, 95% CI = 0.01 to 0.31, *P* = .008) (Fig. 4A). It is evident that in the CRPP group, patients have fewer scars.

### 3.11. Avascular necrosis

Four reports provided data on the incidence of avascular necrosis, and a fixed-effects model was utilized.^[12,15–17]^ No significant heterogeneity was found (*I*^2^ = 2%, *P* = .31). The results indicated no significant difference in the incidence of avascular necrosis between the open group and the closed group (OR = 0.84, 95% CI = 0.09–7.79, *P* = .88) (Fig. 4B).

### 3.12. Delayed union

The incidence of delayed union was recorded in 4 reports,^[13–15,17]^ and only 1 case occurred in the open group in 1 study. A fixed-effects model was applied. The results revealed no significant difference in the occurrence of delayed union between the open group and the closed group (OR = 1.49, 95% CI = 0.06–37.35, *P* = .81) (Fig. 4C).

### 3.13. Discussion

LCFs are a common intra-articular fracture and represent the second most frequent distal humerus fracture.^[4,20]^ In recent years, Song proposed the use of an internal oblique view to facilitate the assessment of LCFs and introduced the Song stage system to better describe the degree and stability of fracture displacement and guide treatment decisions. Concurrently, Song conducted extensive research on CRPP, initially using it to treat Song stage 3 and 4 fractures before conducting further studies on its efficacy for treating Song stage 5 fractures.^[21–23]^

Common complications associated with LCFs in children include superficial and deep infections, joint stiffness, nonunion, delayed union, avascular necrosis, malunion, fishtail deformity, lateral spurring, and growth block.^[1,24–26]^ In the past, surgeons believed that adequate fracture reduction and stable fixation were necessary to reduce the occurrence of these complications.^[27]^ However, some scholars conducted studies on the efficacy of CRPP for treating LCFs and found it to be a suitable treatment option.^[16,21–24,28–30]^ Nevertheless, not all scientists agree with this conclusion, and there is no clear consensus on whether CRPP can effectively treat even the most displaced or unstable LCFs. After all, in the past, LCFs with obvious displacement was required to be anatomically reduced.^[5–9]^

In this meta-analysis, our study compared the efficacy of CRPP and ORPP in treating LCFs. Our main conclusions are as follows: CRPP has a risk of failure during the operation, but it can be converted to ORPP without affecting the treatment of LCFs, as demonstrated prior studies.^[29,30]^ Both CRPP and ORPP can achieve satisfactory elbow function recovery after treatment. Although the excellent rate of functional recovery was lower in the closed group than in the open group, it was not statistically significant. Compared with CRPP, ORPP is associated with a higher risk of unaesthetic scars after the operation, as confirmed by several studies.^[13,15–17]^ CRPP has smaller incision, less soft-tissue loss, and lower scar incidence, which is consistent with the data conclusion. Our study found no significant difference between CRPP and ORPP in terms of superficial infection, deep infection, avascular necrosis, delayed union and other complications. It confirms that CRPP has the same therapeutic effect and prognosis after satisfactory reduction during operation compared with ORPP. In fact, ORPP has a larger surgical incision compared to ORPP. And CRPP had a lower infection rate in many studies,^[13,14,16]^ consistent with our speculation. However, our current research results indicate that there is no significant difference in infection rates between these 2 surgical methods. This result may be related to the relatively small number of included literature.

Based on our current findings, our study still exercise caution when considering ORPP as the first-line treatment for unstable LCFs. Nevertheless, both CRPP and ORPP can achieve favorable clinical outcomes and acceptable complication rates in the treatment of LCFs with complete displacement and rotation. Given the inherent advantages of CRPP, such as a lower risk of visible incision scars and less soft-tissue damage, our study suggest that experienced surgeons continue to consider CRPP for treating LCFs. Of course, it is still a little difficult for young doctors to implement satisfactory reduction effect through CRPP.^[2,18]^

Our study did not find any high-quality RCTs in the existing literature. Therefore, our study included all non-RCTs that met our criteria. However, some of these studies had incomplete data.^[12–17]^ In fact, our study failed to conduct a hierarchical analysis of different types of fractures due to a lack of data. Based on the varying types of fractures observed in those studies, our study consider fracture with displacement exceeding 2 mm as high grade fracture to discuss.

At the same time, findings reveal that the included literature did not describe the evaluation of the joint surface during follow-up, which posed certain difficulties in evaluating complications. And some research data are not provided completely, so our study cannot proceed with further analysis in these areas (follow up, malunion, fishtail deformity, and lateral spurring). In fact, there is limited research data available. To control the type 2 errors, pooled results were calculated using a fixed-effects model for values of *P* > .10 and I^2^ < 50%, or a random-effects model for values of *P* < .10 and I^2^ > 50%. Furthermore, our study only identified 6 relevant studies, and a limited number of primary studies may not provide strong, high-level evidence. Although the 2 studies scored 6 on The NOS,^[12,13]^ their results are consistent with other high-quality studies, so their impact on the overall results is relatively small. Future research should further focus on controlling confounding factors (like gray literature, heterogeneity, and subgroup analysis) and ensuring the completeness of follow-up to improve research quality. Our study excluded non-English literature and gray literature. While these decisions were made after careful consideration, they may introduce selection bias and limit the comprehensiveness of the findings. In the future, our study believe that more RCTs with larger sample sizes and stricter research design are needed to confirm the difference between CRPP and ORPP in treating LCFs. Our next research goal is to investigate whether CRPP treatment failure has additional impacts on patient outcomes.

## 4. Conclusion

While CRPP has a risk of operation failure compared to ORPP, there is no significant difference between the 2 techniques in terms of surgical time, elbow function, superficial and deep infection rates, total infection rate, avascular necrosis or delayed union. Nevertheless, CRPP can significantly reduce the occurrence of unsightly scars. At the same time, CRPP treatment can still be converted to ORPP after failure. Due to limitations in the existing evidence, such as the small number of studies, limited sample sizes, and potential risk of bias, the conclusions drawn may be subject to uncertainty. Our research suggests that qualified closed reduction is a viable option for doctors to treat LCF.

## Author contributions

**Conceptualization:** Zhanchun Li.

**Data curation:** Xinxin Xu, Jicheng Zeng.

**Formal analysis:** Jicheng Zeng.

**Investigation:** Xinxin Xu, Jicheng Zeng.

**Methodology:** Kai Wang, Qiyu Meng, Si Yuan, Jiangtao Shen, Xitao Sun.

**Project administration:** Zhanchun Li, Xitao Sun.

**Supervision:** Qiyu Meng, Zhanchun Li, Xitao Sun.

**Validation:** Xitao Sun.

**Visualization:** Xitao Sun.

**Writing – original draft:** Xinxin Xu, Jicheng Zeng.

**Writing – review & editing:** Xitao Sun.
